# Epicardial Ligation of the Left Atrial Appendage in Octogenarians: Safety and Long-Term Efficacy

**DOI:** 10.3390/jcm14061787

**Published:** 2025-03-07

**Authors:** Karin Nentwich, Nuki Kazaishvilli, Elena Sauer, Artur Berkovitz, Julian Mueller, Sebastian Barth, Thomas Deneke

**Affiliations:** 1 Department of Invasive Electrophysiology, Campus Bad Neustadt, Von Guttenbergstrasse 11, 97616 Bad Neustadt a. d. Saale, Germany; nuki.kazaishvilli@campus-nes.de (N.K.); elena.sauer@campus-nes.de (E.S.); artur.berkovitz@campus-nes.de (A.B.); thomas.deneke@klinikum-nuernberg.de (T.D.); 2 Department of Cardiology, Phillips-University of Marburg, 35043 Marburg, Germany; sebastian.barth@campus-nes.de; 3 Department of Cardiology, Universitäts-Herzzentrum Bad Krozingen, 79189 Bad Krozingen, Germany; julian.mueller@uniklinik-freiburg.de; 4 Department of Cardiology and Imaging, Campus Bad Neustadt, 97616 Bad Neustadt a. d. Saale, Germany

**Keywords:** epicardial ligation, left atrial appendage, octogenerians, stroke prevention, atrial fibrillation

## Abstract

**Introduction:** Due to the aging population, the number of elderly patients with atrial fibrillation and contraindications for anticoagulation due to bleeding complications is growing. After the epicardial ligation of the left atrial appendage (LAA), anticoagulation can be omitted. We present the single-center procedure data and long-term data of octogenarians being treated with LARIAT^®^. **Method:** Out of 145 patients eligible for the epicardial ligation of the LAA, 45 were older than 80 y and included in this analysis. After successful ligation, patients were screened at 6 weeks of follow-up (FUP), at 12 weeks and after 12 months for transesophageal echocardiography (TOE) and clinical events. During long-term FUP, TOE sessions and clinical events for embolic events and death were documented. **Results:** The procedure was successful in 93% of patients, with a mean CHA_2_DS_2_VASC score of 4.6 and HASBLED score of 3.7 and a mean age of 82 y. One major complication occurred, with the laceration of the LAA and surgical closure of the LAA with an Atriclip. The 6-week FUP data were available in 39 patients, with the detection of four leaks (1–3 mm, median 2 mm) and three thrombi; one thrombus occurred at the site of a leak. The 12-week FUP (in 26 patients) showed that three leaks were closed, one leak persisted and one new thrombus developed at the site of the leak. All thrombi were resolved. The 12-month FUP showed the persistent resolution of three thrombi; one thrombus recurred after the withdrawal of the anticoagulant, and no new gap or thrombus could be detected. The long-term FUP (mean 38 months) was documented in 30 patients, with no new gaps and no new thrombi; one patient suffered from a stroke, with a good long-term result of LAA closure in TOE (stroke rate 1%/y, absolute risk reduction of 4.4% to a stroke rate of 5.4% related to the score, relative CHA_2_DS_2_VASC risk reduction of 88%). Eleven patients died: four in the first year of ligation and seven during long-term FUP. **Conclusions:** The epicardial ligation of the LAA for stroke prevention in octogenarians is highly safe and effective. Early TOE FUP is crucial for the detection of thrombi and establishing an optimal anticoagulation regime. No late development of thrombi or gaps can be observed at up to 5 years.

## 1. Introduction

Due to the aging population, patients with atrial fibrillation (AF) and recommendations for anticoagulation for stroke prevention are becoming older. The prevalence of atrial fibrillation in octogenarians is greater than 10% [1]. Age is not just a risk factor for stroke (CHA_2_DS_2_VASC score) but also a risk factor for bleeding (HASBLED score). The mean age and number of patients with the main indication (bleeding complication under NOAK) for the occlusion of the left atrial appendage is growing.

The first large randomized studies showed the non-inferiority of endocardial LAA occlusion with Watchman versus warfarin in terms of stroke, systemic embolism and cardiovascular mortality [2,3,4]. However, the mean ages of the study populations in these first groundbreaking trials were 72.2 y, 74.4 y and 73.4 y. In the Amplatzer trial [5], the mean age was 75.8 y, and it showed a low stroke rate and fewer bleeding events in patients with ASS monotherapy or no anticoagulants. Bleeding complications under low-dose aspirin have also been described by other studies [6,7]. However, age-related fragility and comorbidities lead to the inadequate treatment of these patients with the conservative use of LAA occlusion or full-dose anticoagulation. The ESC Guidelines 2024 recommend LAA occlusion with a C-level IIb, showing the proven non-inferiority of LAA occluders in terms of stroke and non-systemic embolism prevention and significant reductions in bleeding and mortality [8,9]. Procedure-related complications, as well as peridevice leaks (PDL) or thrombus formation, are still an issue of concern for all available LAA closure techniques.

**Figure 1:**
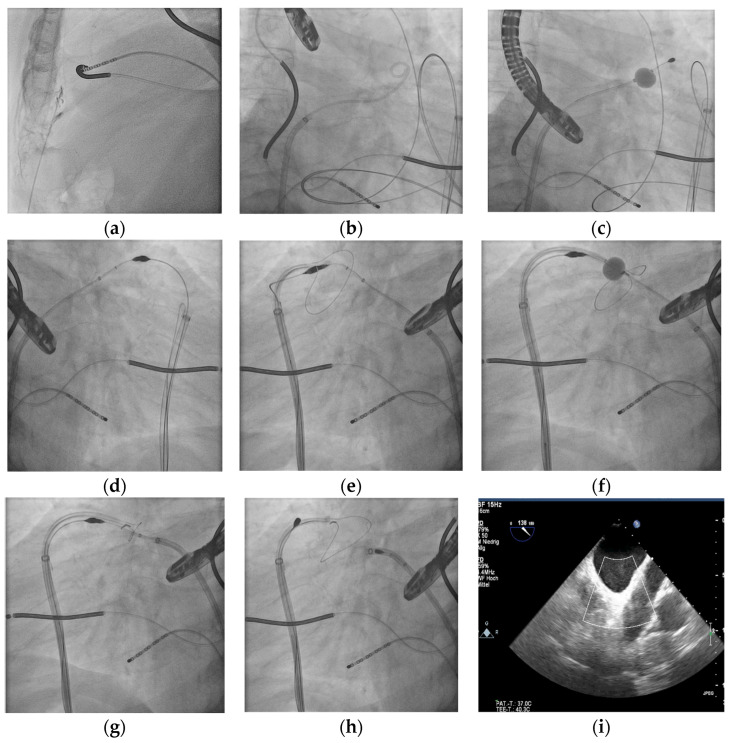
Stepwise approach to the epicardial ligation of the LAA: (**a**) epicardial puncture and insertion of the soft sheet, (**b**) transseptal access with LAA angiography over pigtail catheter, (**c**) endowire position in the anterior lobe of LAA, (**d**) epiwire position with connection of wire magnets, (**e**) opened snare of the LARIAT device passing the LAA, (**f**) epicardial LARIAT device placed in position over LAA along the epiwire, (**g**) ligation of the LAA using the LARIAT snare device, (**h**) release of the snare after tightening the sutures, (**i**) final TOE (130°) with documented complete ligation of LAA.

With the epicardial ligation approach using LARIAT^®^, all anticoagulation may be omitted after the successful closure of the left atrial appendage, as no foreign body is implanted and the appendage is ligated with a suture after puncturing the pericardium (see Figure 1). The first data showed the high efficacy and safety of the LARIAT device [10,11] and non-inferiority to endocardial devices, even with no form of postinterventional anticoagulation [12]. However, the mean ages of the patient cohorts in these trials were 72.1 y, 62.0 y and 73.3 y. Older patients with fragile vessels, as in amyloid angiopathy or angiodysplasia, do not tolerate even ASS. As our center is well trained in epicardial puncture, we started with epicardial ligation using the LARIAT^®^ system as the primary approach in our institution for LAA occlusion in all patients with no contraindications for epicardial puncture. We present our single-center procedure data and long-term data for all octogenarians treated with the LARIAT^®^ device.

## 2. Method

### 2.1. Patient Selection

From November 2016 to July 2023, 230 patients with a CHA_2_DS_2_VASC score > 2 and contraindications for anticoagulation met the criteria for interventional LAA closure. Of these, 145 (59%) were eligible for epicardial ligation (Table 1). All patients were screened with a cardiac CT scan for the evaluation the morphology and size of the LAA. Forty-five patients aged ≥ 80 y were included in this retrospective analysis. Of these, 44 patients had contraindications for anticoagulation due to a history of bleeding and one patient due to repetitive falls. Twenty-three octogenarians with contraindications for the epicardial ligation of the LAA were treated with an endocardial device.

### 2.2. Procedure

After having given informed consent, the patients were sedated with continuous propofol infusion combined with boli of piritramide. After puncturing the pericardium and introducing a soft tip in the pericardial space, a transseptal puncture was performed under TOE guidance [13]. Identifying the anterior lobe in the TOE, combined with the angiogram of the LAA, an endocardial wire with a magnet at its end was placed in the LAA anterior lobe. Via epicardial access, a second magnet-attached wire was introduced and connected to the endocardially placed magnet. Over the connected magnet wires, the snare was advanced over the LAA to the LAA neck. The optimal closing position was confirmed by an angiogram, balloon insufflation and TOE guidance. After closing down the snare, the complete capture of the LAA was ensured via TOE. The suture was released. After 2 tightenings of the suture with a tension device, the result of the ligation was confirmed by TOE for central gaps or lost lobes. The suture was cut and a pigtail drain was placed in the pericardial space. See the illustration in Figure 1. The patient was monitored in the ICU for 24 h. For the prevention of pericarditis, treatment with colchicum was started before the procedure and continued for 6 weeks. All patients were discharged without any form of anticoagulation, except for those with known coronary heart disease, being treated with 100 mg acetyl acid/d.

The patients’ data and procedural data were documented during their hospitalization. The presence of minor complications necessitated nominal therapy or no treatment with longer observation. Major complications were defined as a medical condition that significantly impacted patients’ care, treatment and resource utilization.

### 2.3. Follow-Up

Transesophageal FUP was scheduled at 6 and 12 weeks and 12 months. Leaks were defined as a central gap ≥ 1 mm, and the size and location of thrombus formation were described. Clinical FUP (death, embolic event/stroke or readministration of anticoagulation) was documented during all TOE sessions and one every 12 months.

The procedural data, confounders and FUP data were documented in SPSS 2023 and are expressed as the mean ± standard deviation (SD) for continuous variables or as numbers and percentages for categorical variables. No adjustments were performed. The Kaplan–Meier curve was created with SPSS 2023.

## 3. Results

### 3.1. Procedure

Epicardial ligation using the LARIAT system was successfully performed in 43 patients (93.3%) (24 males). The mean age was 82.6 y (range 80–90 y), the mean CHA_2_DS_2_VASC score was 4.6 and the mean HASBLED score was 3.7. For more details of the patients’ characteristics, see Table 2. The mean procedure time was 87.4 ± 27 min, and the mean radiation time was 16.5 ± 8 min. In two patients, pericardial adhesions prevented successful epicardial ligation. All patients exhibited terminal renal failure under dialysis. One major complication occurred with the laceration of the LAA, requiring the emergency surgical closure of the LAA with an Atriclip. Four minor complications were documented: two patients with prolonged bleeding over the draining pigtail with optimizing hemostasis and two patients who developed Dressler syndrome, requiring steroids (Table 3).

### 3.2. TOE and Clinical FUP

At the 6-week FUP, data from 39 (86%) patients were available. Four leaks (1–3 mm, median 2 mm) and three thrombi were detected, with one thrombus occurring at the site of a leak. All patients with thrombi were anticoagulated. One patient died 4 weeks after the procedure due to cerebral bleeding and one patient due to COVID-related pneumonia.

A 12-week FUP was performed in 26 (57.7%) patients. One leak persisted and three previously detected leaks spontaneously closed. The thrombi detected at 6 weeks were resolved under anticoagulation therapy. OAC therapy was discontinued in these patients. There was one new thrombus detected at the site of a previously detected leak. No new leaks were detected. One patient died because of recurrent cerebral bleeding and one patient with unknown circumstances.

A 12-month FUP was performed in 17 (37.7%) patients. All prior documented leaks spontaneously closed. There were no new leaks or thrombi detected. Thrombi did not reoccur after the withdrawal of anticoagulation in three previously detected cases of thrombi. One thrombus reoccurred after successful resolution with 6 weeks of OAC therapy. No embolic event occurred in this patient. The patient with the recurrent thrombus was treated with half NOAK therapy continuously. No further deaths occurred.

**Figure 2:**
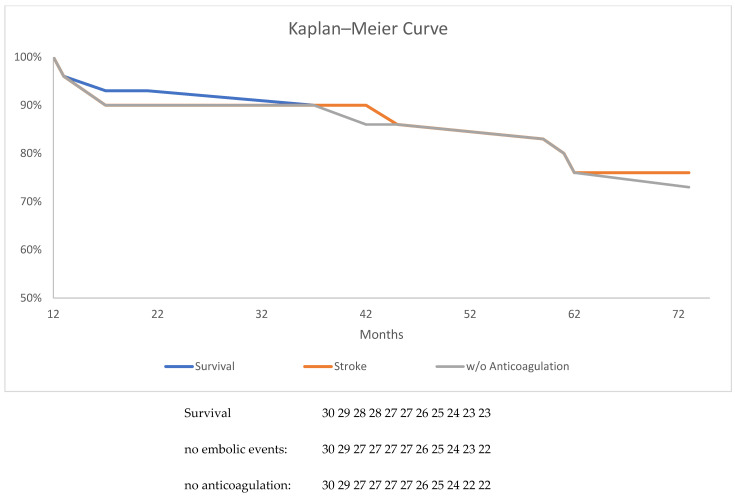
Kaplan–Meier curve of survival, embolic events and readministration of anticoagulation in percent.

Long-term FUP was performed in 30 (71.1%) patients for more than 1 year (Figure 2). The mean FUP interval was 38.4 ± 18 months (range 13–73 months). TOE was available in 21 patients; no new leaks or new thrombi were detectable. One patient developed a stroke, with a good long-term result of the ligation in TOE (Table 4).

The stroke rate could be calculated as 1.0%/y. According to the estimated stroke rate of 5.4%/y, with a CHA_2_DS_2_VASC score of 4.6, this presents an absolute risk reduction of 4.4% and a relative risk reduction of 88%.

Seven patients died. The mortality rate was calculated as 23%, with a mean mortality rate of 7.1% per year. One patient was anticoagulated with NOAK due to the recurrent development of thrombi.

## 4. Discussion

Our retrospective analysis after the epicardial ligation of the LAA shows high safety and efficacy in an older patient population. The incidence of thrombus formation in the first 6–12 weeks was high at 9.3% in this high-risk group; however, no new thrombi could be detected after 12 weeks, all thrombi were resolved by anticoagulation, and there were no embolic events. There was no late thrombus formation noted for up to 5 years. The closure rate with no leaks was 92.7% at 6 weeks, with the spontaneous closure of 9.3% of the leaks noted at 6 weeks, as seen at the follow-up at 12 weeks. No clinical embolic events occurred in the first 12 months, and one patient developed a stroke after 21 months, but they had a good long-term LAA closure result by TOE. The mortality rate of 7% after successful epicardial ligation is lower than expected for this age.

Although NOACS exhibits better outcomes in octogenarians in terms of stroke, intracranial bleeding and mortality versus warfarin, major bleeding and GI bleeding remain a clinical issue [14]. Due to fragility and its associated risk of complications, older patients with atrial fibrillation lack optimal treatment in a real-world setting. Gafoor et al. showed that the success rate, safety and 1-year efficacy were 90.1%, 3.9% and 97.3% with various techniques of LAA occlusion [15]. Another trial on a combined procedure with AF ablation and Watchman implantation showed high safety in octogenarians compared to younger patients, with just one major bleeding event, a 2.1% stroke rate/y, a peridevice leak rate of 20% and one thrombus after 3 months [16]. A retrospective analysis of data from octogenarians in the Protect AF and Prevail trials demonstrates the same efficacy and safety of LAA occlusion with the Watchman device as in patients of a younger age [17]. However, one meta-analysis on endocardial LAA occlusion found higher periprocedural mortality, major bleeding events and pericardial effusion in the elderly, with the same implantation success rate and stroke rate [18].

Older patients exhibit much higher mortality rates, irrespective of LAA occlusion. The mortality rate and bleeding rate were higher in the elderly at the 1-year follow-up in the meta-analysis mentioned above [18]. No studies have shown a significant benefit for mortality in octogenarians over younger patients after LAA occlusion [16,17]. The mortality rate in the first year after implantation varies from 3.0 to 15.5% [19]; in our cohort, it was 9.5% (all non-procedure-related). Poor cost-effectiveness and even futility could be suggested. Mesnier et al. [20] analyzed a cohort of 807 patients with a mean age of 76 ± 8 y, with 125 early deaths (15.5%) within the first year after implantation. A higher age, lower body mass index, impaired glomerular filtration, prior heart failure and diabetes were independent risk factors for early death. The combination of up to three risk factors resulted in a mortality risk of >50%. This might limit the potential beneficial effect of the procedure. The careful evaluation of patients recommended for LAA occlusion is crucial. Further insights will be provided by the ongoing trial Frail LAAC (NCT05257954).

The thrombus rate was high in our cohort at the 6-week FUP (6.9%), declining after 12 weeks to 2.3%, with the resolution of all thrombi under NOAK. There was no post-LAA ligation anti-thrombotic therapy except for ASA in patients with coronary artery disease. No embolic events occurred in patients with thrombi. It is well known that a higher age is a risk factor for thrombus formation after LAA occlusion, as well as many other factors [21,22]. The incidence of DRT with endocardial devices has been reported to be 3.8 to 7.2% [23,24]. Embolic events with endocardial device-related thrombi have been established [25]. In contrast to endocardial devices [26], there was no new or late thrombus development during the FUP over more than 5 years. No clinical embolic events were noted in our cohort. Previous studies with LAA ligation had thrombus rates of 1–2% [11,27]. Potential factors contributing to increased thrombus formation are the increased age and CHA_2_DS_2_VASC scores of this population. For epicardial ligation, it seems to be crucial to detect thrombi in the early state to ensure optimal medical anticoagulation and the prevention of clinical events.

Another issue of concern is peridevice leaks after the closure of the LAA. For the endocardial approach, the closure of the LAA with PDL < 5 mm has been defined as successful. Meanwhile, data have been published showing that any gap size can be associated with stroke and may not be benign [28,29]. In all of our patients, the complete closure of the LAA was documented in the procedure-related TOE. At the 6-week FUP, four central leaks (9.3%) were noted, all smaller than 4 mm, with a median of 2 mm. All occurred at the beginning of our learning curve, with the careful tightening of the sutures in frail patients. Three leaks closed spontaneously within 12 weeks after LAA ligation. Although leaks have been associated with thromboembolic events with endocardial devices and reported in the surgical LAA exclusion literature, the significance of leaks following LAA ligation is unclear, since only one of the noted thrombi was seen at the site of a leak. Since leaks associated with LAA ligation are central leaks, such leaks can be easily closed with endovascular devices [30]. The spontaneous closure of PDLs of endocardial devices has also been shown to decrease from 40.9% to 32.1% in the Protect trial [31]. However, a new PDL could also be detected with Watchman in 8.3% and with Amulet in 4.2% [32]. This phenomenon has not been described before for epicardial ligation and could not be reported in this study.

Although octogenarians have higher CHA_2_DS_2_VASC scores and a higher risk of embolic events, significant reductions in embolic events could not be observed when compared with a younger patient cohort in any trial with endocardial LAA occlusion [17,18,33]. In our cohort, we reported just one stroke with a mean FUP interval of 38 months (range 2–73 months), resulting in a stroke rate of 1%/y. This is lower than other reported stroke rates (2.2%/y in the Prevail/Protect AF trials in the elderly and 2.1%/y in the combined ablation and LAA occlusion cohort) and lower than the expected stroke rate of 5.4% according to the CHA_2_DS_2_VASC score of 4.6. This indicates an absolute risk reduction of 4.4% and a relative risk reduction of 88%. It can be summarized that the closure of the LAA in octogenarians is highly effective in terms of stroke reduction.

Nevertheless, the considerable incidence of bleeding complications after endocardial LAA occlusion can be observed in older patients [18,34]. It should be noted that post-intervention anticoagulation with DAPT and ASA after 3 months might be harmful for older patients [7,35]. Epicardial ligation might be the preferred technique for older patients as any form of anticoagulation can be omitted after closure. In our cohort, all patients were discharged without anticoagulation, except for those with known coronary heart disease, being treated with 100 mg ASA.

## 5. Limitations

This was a retrospective observational study in a real-world scenario with a small sample size, and there were limitations due to the patients’ compliance, like missed FUP, a lack of medication intake and low motivation for medical control. Some patients developed heart failure or ACS and were treated with valve repair or stent implantation, meaning a change in anticoagulation. Some patients received specific heart failure care and this might have improved their outcomes. Selection bias was possible due to the specific inclusion and exclusion criteria for this patient cohort.

## 6. Conclusions

These data show the high safety and efficacy of epicardial ligation using the LARIAT system^®^ in octogenarians, with a low embolic event rate at up to 5 years of FUP. Early FUP after 6 weeks is crucial in detecting thrombus formation and ensuring an optimal anticoagulation regimen. Thrombi show a high resolution rate, and all leaks were endothelialized without any clinical events. No development of new thrombi or gaps over a long-term period of up to 5 years was observed.

Randomized trials comparing endocardial LAA occlusion with epicardial ligation are warranted to confirm these observations.

## Figures and Tables

**Table 1:** Screening failures for epicardial ligation of the LAA with LARIAT.

Clinical Contraindications	Anatomical Contraindications
History of cardiac surgery	Superiorly or backwards orientated LAA with the anterior lobe behind the pulmonary trunk
Renal failure with dialysis	Left rotated heart
Pectus excavates	LAA width > 50 mm
History of thoracic radiation	Multiple lobes with different orientations and wider distance than 50 mm
NYHA IV classification	Adipositas BMI > 50
Planned cardiac surgery with surgical LAA resection	Thrombus in LAA
Adhesions (uremic pericarditis)	

**Table 2:** Patients’ characteristics.

Characteristic	n = Number (%)
Gender (male)	24 (53.3%)
Mean age (y)	82.6 ± 2
CHADSVASC score	4.7 ± 1
HASBLED score	3.6 ± 0.5
Ejection fraction (%)	60 ± 10.5
Coronary heart disease	24 (53.3%)
Prior pulmonary vein isolation	4 (8.8%)
Prior stroke	7 (15.5%)

**Table 3:** Procedure characteristics.

Characteristic	n = Number (%)
Duration in min	87 ± 27
Radiation time in min	16.9 ± 14
Successful ligation	95.5%
Major complications	1
Minor complications	2

**Table 4:** Findings depending on the FUP timing; * no new thrombus or gap detected.

	6-Weeks FUP	12-Week FUP	12-Month FUP	Mean 38-Month FUP
Gap	4	1	0 *	0 *
Thrombus	3	1	0 *	0 *
Embolic event	0	0	0	1
Death	2	2	0	7

## Data Availability

The raw data supporting the conclusions of this article will be made available by the authors on request.

## Abbreviations

AF	atrial fibrillation
LAA	left atrial appendage
FUP	follow-up
TOE	transesophageal echocardiogram
NOAK	novel oral anticoagulation
PDL	peridevice leak
ICU	intensive care unit
SD	standard deviation
